# Weekly injections of Hylan G-F 20 delay cartilage degeneration in partial meniscectomized rat knees

**DOI:** 10.1186/s12891-016-1051-6

**Published:** 2016-04-27

**Authors:** Katsuaki Yanagisawa, Takeshi Muneta, Nobutake Ozeki, Yusuke Nakagawa, Mio Udo, Ryusuke Saito, Hideyuki Koga, Kunikazu Tsuji, Ichiro Sekiya

**Affiliations:** Department of Joint Surgery and Sports Medicine, Graduate School of Medicine, Tokyo Medical and Dental University, Tokyo, 113-8510 Japan; Center for Stem Cell and Regenerative Medicine, Tokyo Medical and Dental University, 1-5-45 Yushima, Bunkyo-ku, Tokyo, 113-8510 Japan; Department of Cartilage Regeneration, Graduate School of Medicine, Tokyo Medical and Dental University, Tokyo, 113-8510 Japan

**Keywords:** Hyaluronan, Hylan G-F 20, Partial meniscectomy, Osteoarthritis, Rat

## Abstract

**Background:**

Cross-linked hyaluronan—also called Hylan G-F 20—is a medical device developed to treat osteoarthritis of the knee. However, it is still controversial whether Hylan G-F 20 has a cartilage protective effect in trauma-induced osteoarthritis. We investigated whether Hylan G-F 20 delayed osteoarthritis progression in a partial meniscectomized rat model.

**Methods:**

Lewis rats were used for the experiments. The anterior medial meniscus was resected at the level of the medial collateral ligament in both knees. From 1 week after the surgery, 50 μl of Hylan G-F 20 was injected weekly into the left knee and phosphate buffered saline was injected into the right knee. Cartilage was evaluated for macroscopic findings, histology with safranin-o, and expression of type II collagen at 2, 4, and 8 weeks. Synovitis was also evaluated, and immunohistochemical analysis was performed for ED1.

**Results:**

Macroscopic findings demonstrated that India ink positive area, representing fibrillated cartilage, was significantly smaller in the Hylan G-F 20 group than in the control group at 2, 4, and 8 weeks (*n* = 5). There were no significant differences in osteophyte score between the Hylan G-F 20 group and the control group at 2, 4, and 8 weeks.

Histologically, the cartilage in the medial tibial plateau was destroyed at 8 weeks in the control group, while type II collagen expression was still observed at 8 weeks in the Hylan G-F 20 group. OARSI score for cartilage histology was significantly lower in the Hylan G-F 20 group than in the control group at 4 and 8 weeks (*n* = 5). There were no significant differences in synovial cell number or modified synovitis score between the Hylan G-F 20 group and the control group at 2, 4, and 8 weeks (*n* = 5). In the Hylan G-F 20 group, foreign bodies surrounded by ED1 positive macrophages were observed in the synovium.

**Conclusion:**

Weekly injections of Hylan G-F 20 starting 1 week after surgery delayed cartilage degeneration after meniscectomy in a rat model. Synovitis induced by meniscectomy was not alleviated by Hylan G-F 20. Insoluble gels were observed in the synovium after the Hylan G-F 20 injection.

## Background

Osteoarthritis (OA) is one of the most common musculoskeletal diseases and causes joint pain and disability in daily life. Hundreds of millions of people are suffering from the disease worldwide [1] and the prevalence of OA is increasing as the aging population increases [2]. Management of OA is critical from the viewpoint of medical cost, and many efforts have been made to prevent the progression of OA; however, it is still difficult to overcome this pathology [3].

Hyaluronan is a constitution of cartilage and synovial fluid, and contributes to joint homeostasis [4]. In OA, the synovial fluid is less viscous and both the concentration and molecular weight of hyaluronan in synovial fluid decrease [5]; therefore, administration of exogenous viscous hyaluronan may improve these problems. In the synovial fluid of the normal joint, the molecular weight of hyaluronan is 2000–7000 kDa [6–8]. Cross-linked hyaluronan, Hylan G-F 20, is a medical device developed to treat OA of the knee and an elastoviscous high molecular weight fluid derived from chicken combs. Hylan G-F 20 consists of 80 % Hylan A, a soluble high molecular weight, and 20 % Hylan B, an insoluble gel. Hylan A has an average molecular weight of approximately 6000 kDa and Hylan B is a hydrated gel [9]. It is reasonable to administrate hyaluronic acid with a molecular weight similar to that of the normal joint into OA knees.

In clinical situations, Hylan G-F 20 showed a significant reduction in pain for OA patients [10, 11]. One report demonstrated the effectiveness of Hylan G-F 20 for delaying OA progression [12], while another report showed opposite results [13]. It is still controversial whether Hylan G-F 20 has a cartilage protective effect. In animal studies, Hylan G-F 20 was investigated in a rabbit anterior cruciate ligament transection model, in which Hylan G-F 20 was effective in delaying OA progression [14–16]. We already reported that partial meniscectomy induced OA progression in rats and that this is a useful OA model to examine therapeutic effects [17–19]. Here, we investigated whether Hylan G-F 20 delayed OA progression in a partial meniscectomized rat model.

## Methods

### Animals

This study was approved by the Animal Experimental Committee of Tokyo Medical and Dental University. A total of 30 10-week-old wild-type male Lewis rats (Charles River, Kanagawa, Japan) were used for the experiments. They were fed under standard conditions that changed every week (12-h light:12-h dark cycle, room air temperature 22–24 °C, 2 rats per cage). The weight ranged from 280 to 296 g.

### Experimental OA model

**Fig. 1:**
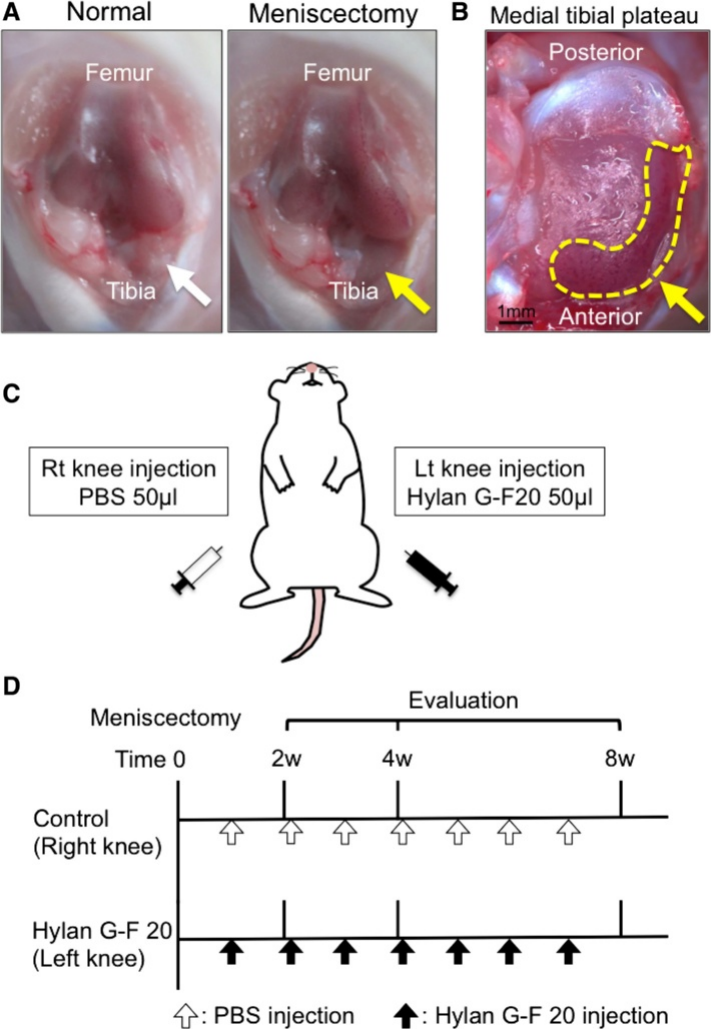
Experimental set up. **a** Knee joint before and after meniscectomy. Medial meniscus is indicated with white arrow, and meniscal defect with yellow arrow. **b** Tibial plateau after meniscectomy. Meniscus defect is indicated with yellow dot. **c** Study schema. **d** Study protocol

Rats were anesthetized by isoflurane inhalation and intraperitoneal injection of 2,2,2-tribromoethanol (Avertin®; Sigma-Aldrich, Mo.). Both the right and left knee joints received surgery. After medial parapatellar incision and lateral dislocation of the patellar tendon, the medial meniscus was exposed (Fig. 1a). Then the anterior insertional ligament of the medial meniscus was transected to dislocate the medial meniscus anteriorly, and the medial meniscus was resected at the level of the medial collateral ligament (Fig. 1b) [17–19]. The joint capsule and the overlying fascia were subsequently sutured. The rats were allowed to walk freely in their cages.

### Intra-articular injections of Hylan G-F 20

One week after the surgery, the left knee joint had intra-articular injections of 50 μl [20] of Hylan G-F 20 (molecular weight; 6000 kDa, Synvisc**®**; Teijin Pharma Ltd., Tokyo, Japan) every week (Fig. 1c). The right knee joint received weekly injections of phosphate buffered saline (PBS) as a control (Control Group). The knees were evaluated at 2 (*n* = 10 for each group), 4 (*n* = 10 for each group), and 8 (*n* = 10 for each group) weeks after the surgery (Fig. 1d).

### Macroscopic observation

For the evaluation of the medial tibial plateau cartilage, India ink was brushed on the cartilage surface using a swab and left for 10 s; then the surface was washed with a new swab to remove the ink from the intact cartilage area. Macroscopic pictures were taken using an Olympus MVX10 microscope (Olympus, Tokyo, Japan) on a dedicated medical photography platform.

**Fig. 2:**
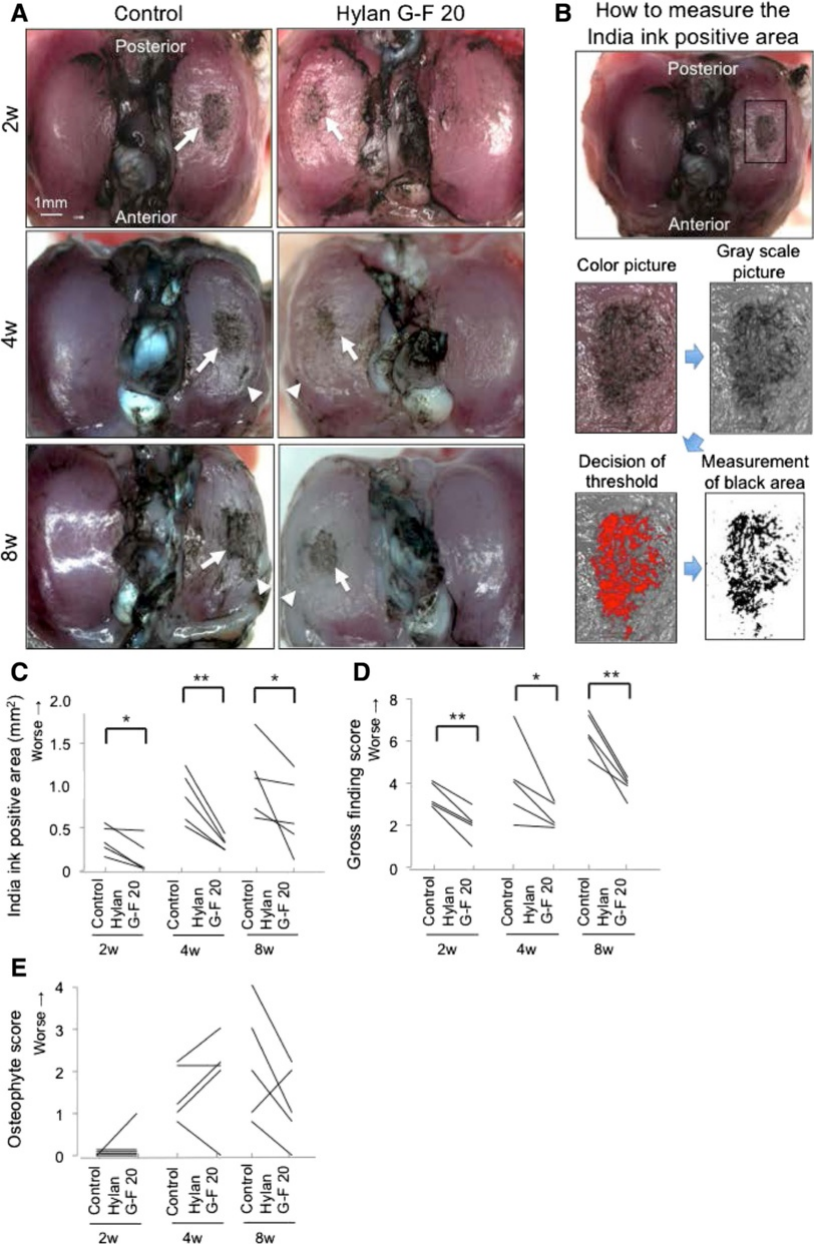
Analyses of macroscopic observations for the medial tibial cartilage. **a** Macroscopic features stained with India ink. Cartilage erosion is indicated with arrow. Osteophyte is indicated with arrowhead. **b** Method for measurement of India ink positive area of the medial tibial plateau. A boxed area to measure India ink positive area was fixed, the fixed area was gray-scaled, the threshold for India ink positive area was decided, and India ink positive area was quantified. **c** Quantification of India ink positive area (*n* = 5, *p < 0.05, **p < 0.01 by Paired *t*-test). **d** Gross finding score (*n* = 5, *p < 0.05, **p < 0.01 by Paired *t*-test). **e** Osteophyte score (*n* = 5)

India ink positive area on the medial tibial condyle was quantified using the software Image J (National Institutes of Health, Bethesda, Maryland: Fig. 2b).

The gross finding score in the articular cartilage was graded as follows: grade 1, intact articular surface; grade 2, minimal osteophyte; grade 3, over spur formation; grade 4, width of erosion area 0 to 0.5 mm; grade 5, width of erosion area 0.5 to 1 mm; grade 6, width of erosion area 1 to 1.5 mm; grade 7, width of erosion area 1.5 to 2.0 mm; grade 8, width of erosion area >2.0 mm [21].

The osteophyte score was graded as follows: grade 0, normal around the medial tibia; grade 1, osteophyte formation 1/3 around the medial tibia; grade 2, osteophyte formation halfway around the medial tibia; grade 3, osteophyte formation 2/3 around the medial tibia; grade 4, osteophyte formation all-around the medial tibia.

### Histological evaluation of the medial tibial cartilage

The tibial plateau (*n* = 5 for each group) was fixed in 4 % paraformaldehyde for 7 days, decalcified in 20 % EDTA solution for 21 days, then embedded in paraffin wax. The specimens were sectioned in the sagittal plane at 5 μm and stained with safranin-o and fast green. Histologic sections were visualized using an Olympus BX53 microscope (Olympus, Tokyo, Japan). Cartilage degeneration was evaluated using the Osteoarthritis Research Society International (OARSI) score, on a scale of 0–24 points [21].

### Immunohistological analysis of the medial cartilage

Paraffin-embedded sections were deparaffinized in xylene, rehydrated in graded alcohol, and washed with PBS. Then the samples were pretreated with 0.4 mg/ml proteinase K (Dako) in Tris HCl buffer for 15 min; then endogenous peroxidases were quenched using 0.3 % hydrogen peroxidase in methanol for 30 min. Primary antibodies (human anti–type II collagen; 1:500 dilution [Daiichi Fine Chemical]) were applied to sections and incubated at room temperature for 1 h. The sections were incubated in the secondary antibody biotinylated horse anti-mouse IgG for type II collagen (1:200 dilution; Vector) for 30 min at room temperature. Immunostaining was detected with Vectastain ABC reagent (Vector) followed by diaminobenzidine staining. The sections were counterstained with hematoxylin.

### Histological and immunohistological analysis of synovium

The whole knee joint (*n* = 5 for each group) was fixed in 4 % paraformaldehyde for 7 days, decalcified in 20 % EDTA solution for 21 days, then embedded in paraffin wax. The specimens were sectioned in the sagittal plane at 5 μm and stained with hematoxylin and eosin. Histologic sections were visualized using an Olympus BX53 microscope. The number of synovial cells was counted within the 100 μm of the randomly selected three regions of interest, and the average number was evaluated. Synovitis was also evaluated using a modified synovitis score, on a scale of 0–6 points [22]. These quantification evaluations were performed to examine whether Hylan G-F 20 improved synovitis; therefore, the area where foreign bodies were observed in the Hylan G-F 20 group was excluded.

The monoclonal mouse anti-rat ED1 antibody [23] (1:400; Abcam, Cambridge, UK) was used for visualization of macrophage. For ED1, the sections were placed in 4 % paraformaldehyde for 15 min, immersed in sodium citrate buffer (Dako, Carpinteria, CA), placed in hot water at 95 °C for 20 min, and then endogenous peroxidases were quenched using 0.3 % hydrogen peroxide in methanol for 30 min. Primary antibodies for ED1 were applied to sections and kept overnight at 4 °C. After extensive washes with PBS, the sections were incubated in the biotinylated horse anti-mouse IgG for ED1 for 30 min at room temperature. Immunostaining was detected with Vectastain ABC regent (Vector, Burlingame, CA) followed by diaminobenzidine staining. The sections were counterstained with hematoxylin.

### Statistical analysis

StatView 5.0 (SAS Institute, Cary, NC) was used for statistical analyses. Paired *t*-test was performed for the analyses. *P* values less than 0.05 were considered significant.

## Results

### Hylan G-F 20 delayed cartilage degeneration after meniscectomy

Macroscopically, in the control group, India ink positive area was already observed at the medial tibial cartilage 2 weeks after partial resection of the medial meniscus, and enlarged medially at 4 and 8 weeks (Fig. 2a). In the Hylan G-F 20 group, India ink positive area was also observed but appeared to be smaller in each period. Quantification analysis (Fig. 2b) demonstrated that India ink positive area was significantly smaller in the Hylan G-F 20 group than in the control group at 2, 4, and 8 weeks (Fig. 2c). Gross finding score was also lower in the Hylan G-F 20 group than in the control group at each period (Fig. 2d). There were no significant differences in osteophyte score between the Hylan G-F 20 group and the control group at 2, 4, and 8 weeks (Fig. 2e).

**Fig. 3:**
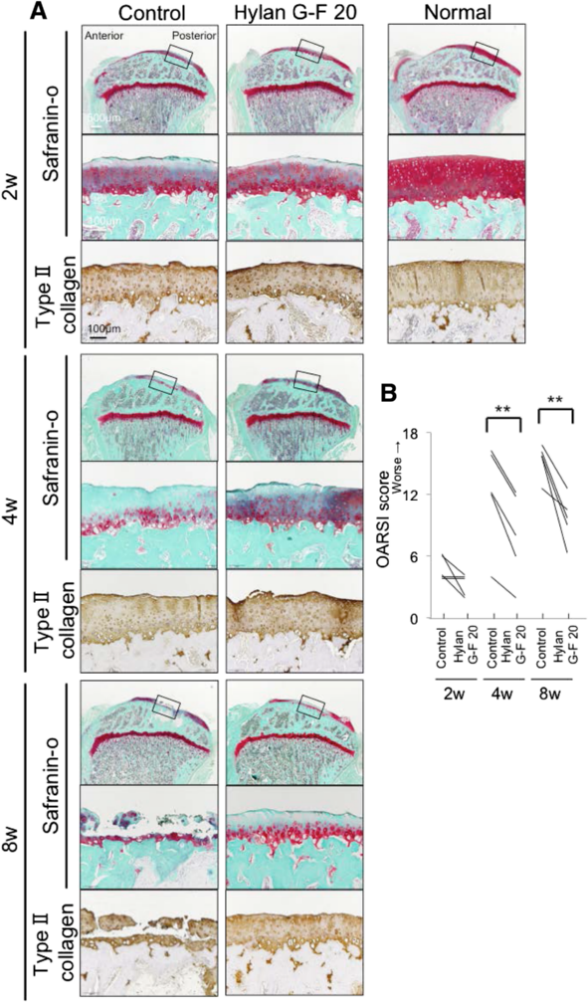
Analyses of histological observations for the medial tibial cartilage. **a** Sagittal sections stained with safranin-o and immunostained with type II collagen. An 18-week-old rat was used as a normal. Boxed areas in the upper panels are shown at a higher-magnification view in the middle panels. **b** OARSI score (*n* = 5, **p < 0.01 by Paired *t*-test)

In the control group, stainability of safranin-o in the medial tibial plateau decreased at 2 weeks, further decreased at 4 weeks, and the cartilage was destroyed at 8 weeks (Fig. 3a). In the Hylan G-F 20 group, cartilage degeneration appeared to be milder than in the control group in each period and type II collagen expression was still observed at 8 weeks. OARSI score was significantly lower in the Hylan G-F 20 group than in the control group at 4 and 8 weeks (Fig. 3b).

### Synovitis induced by meniscectomy was not alleviated by Hylan G-F 20

**Fig. 4:**
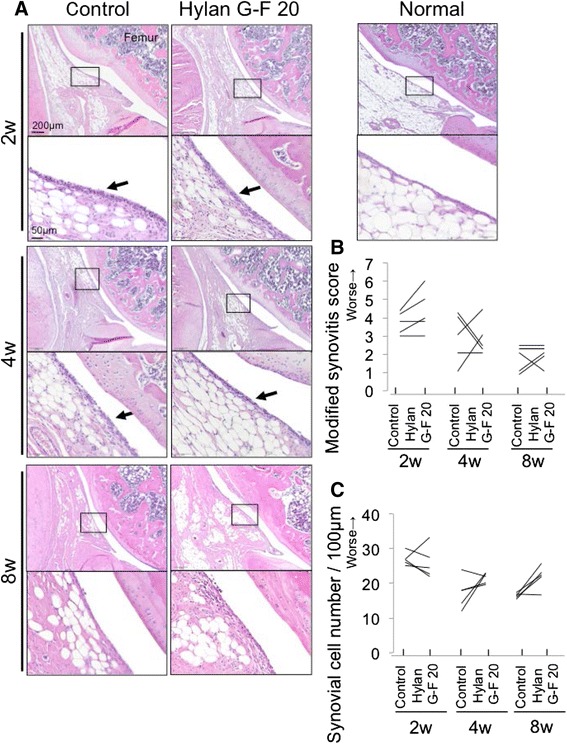
Analyses of histological observations for the infrapatellar fat pad. **a** Sagittal sections stained with hematoxylin and eosin. An 18-week-old rat was used as a normal. Boxed areas in the upper panels are shown at a higher-magnification view in the lower panels. Arrow indicates increased number of synovial cells. **b** Modified synovitis score for infrapatellar fat pad. **c** Synovial cell number/100 μm synovium

The infrapatellar fat pad in the normal knee joint was covered with a single layer or a few layers of synovial cells (Fig. 4a). Thickness of synovial lining layers increased 2 weeks after meniscectomy, and then decreased in both groups. There were no significant differences in synovial cell number or modified synovitis score between the Hylan G-F 20 group and the control group at 2, 4, and 8 weeks (Fig. 4b, c).

### Foreign bodies were observed in the synovium after the Hylan G-F 20 injection

**Fig. 5:**
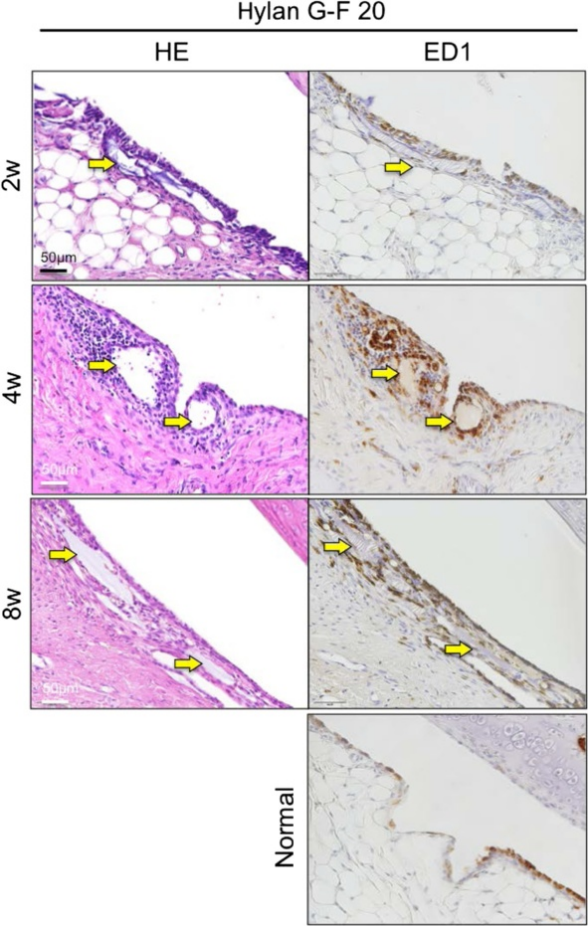
Immunohistochemical analysis of infrapatellar fat pad after the surgery in the Hylan G-F 20 group. Sagittal sections stained with hematoxylin and eosin, and immunostained with ED1. An 18-week-old rat was used as a normal. Yellow arrow indicates gel in the synovium

In the Hylan G-F 20 group, foreign bodies were observed in the synovium at the infrapatellar fat pad in 3 knees at 2 weeks, 4 knees at 4 weeks, and 4 knees at 8 weeks among 5 knees. They were surrounded with multilayered synovial cells positive for ED1 (Fig. 5).

## Discussion

We demonstrated that intra-articular injections of Hylan G-F 20 delayed cartilage degeneration in a meniscectomized rat OA model. Among the surgically induced animal models of OA, the menisci resection model has been widely used [24]. In this study, we removed only the anterior part of the medial meniscus. Though total meniscus resection models may be more popular [25, 26], they seem to be highly invasive and complicated. In our current model, the medial collateral ligament could be preserved, and we could complete this model with lower invasiveness, with more ease, and with higher reproducibility. We previously used this model in rats [17, 18, 27, 28], rabbits [29] and pigs [30]. This model was also used by other groups [24, 26]. Though our model mimics limited pathological conditions, it was useful to investigate the effectiveness of treatment for OA.

Several reports described an anti-inflammatory effect of hyaluronan injected into the knee joints [31, 32]. Smith et al. injected 500–700 kDa hyaluronan intra-articularly in an ewe meniscectomy model and reduced fibrosis [33]. Tang et al. reported that fibrous change of the infrapatellar fat pad due to strenuous running exercise was inhibited by 800 kDa hyaluronan injection in a rat model [34]. In this study, Hylan G-F 20 did not improve synovitis induced by meniscectomy. Furthermore, no evidence was shown for the anti-inflammatory effect of Hylan G-F 20. This suggests that Hylan G-F 20 delayed cartilage degeneration not by an anti-inflammatory effect, but through refinement of lubrication between the articular cartilage of the medial femoral condyle and that of the medial tibial plateau after removal of the anterior medial meniscus.

We tested both Hylan G-F 20 and PBS alone in the same rat, with each knee receiving a different treatment. The main reason for this was that we wanted to exclude inter-animal variability and investigate the effect of Hylan G-F 20 on osteoarthritis progression in a stricter manner. Additionally, we could cut the number of rats in half, in comparison to a study design in which either Hylan G-F 20 or PBS was tested in a rat. One concern is that injection of Hylan G-F 20 into the knee may affect the contralateral knee by spreading via systemic circulation. However, this effect will be negligible because the concentration of hyaluronan in blood hardly increased after intraarticular injection of Hylan G-F 20 [35].

Before this experiment, we had expected that Hylan G-F 20 might promote meniscus regeneration, because Kobayashi et al. reported that injection of 800 kDa hyaluronan increased the size of the meniscus in a massive rabbit meniscectomy model [24]. Contrary to our expectation, we did not find any difference of the medial meniscus in the Hylan G-F 20 group and the control group (data not shown); however, a small amount of regeneration occurred naturally after meniscectomy in rats [17–19].

In clinical situations, Hylan G-F 20 is injected only 3 times every week [36]. In our protocol, Hylan G-F 20 was injected every week and 7 times to evaluate knees at 8 weeks. This is different from the protocol recommended in clinical situations. We performed weekly injections because we want to compare Hylan G-F 20 and another hyaluronan, which is injected every week in clinical situations. We are going to report this comparative study in another paper.

As one possible negative effect of Hylan G-F 20, foreign bodies surrounded with multilayered macrophages were observed in the synovium. This was possibly because Hylan B, an insoluble gel, was taken up by synovium and synovitis occurred locally. Foreign bodies in the synovium were previously reported in other animal studies [37] and in clinical studies [38]. Sasaki et al. demonstrated the subcutaneous and intramuscular induction of a delayed foreign body granulomatous inflammation in guinea pig and rabbit models [39, 40]. Chen et al. reported that inflammation worsened when Hylan G-F 20 was injected into a patient with hydrarthrosis [38]. Granulomatous inflammation within the knee has also been described by Zardawi [41]. Attention must be paid to a foreign body reaction in clinical use.

There are some limitations in the present study. First, we injected Hylan G-F 20 1 week after the surgery when the cartilage degeneration had not yet occurred. Therefore, repetitive injections of Hylan G-F 20 were effective for the prevention of the cartilage degeneration, but we did not identify that this had the same effect in the established OA model. We should explore this in the next study. Second, the molecular mechanism of the chondroprotective effect was not identified from our study. Synovial inflammation was not attenuated by injections of Hylan G-F 20, which indicates the direct effect of cartilage protection. However, we should identify the molecular mechanism of the chondroprotective effect of Hylan G-F 20. In addition, we used young rats in this study; therefore, we should take this situation into consideration when we apply this data to humans. Third, we used PBS as a control. Each 2.25 mL syringe of Hylan G-F 20 contains 16 mg of Hylan polymers (hylan A + hylan B), 17 mg of sodium chloride, 0.32 mg of disodium hydrogen phosphate, 0.08 mg of sodium dihydrogen phosphate monohydrate, and 2.0 mL of water. Additionally, the clinical trial for Hylan G-F 20, i.e., physiological saline, was used as a control [42]. These results suggest that physiological saline was better than PBS as a control in our current study. However, PBS was often used as a control in animal studies to examine the effect of hyaluronan [24, 43, 44]. The use of PBS for the control is not uncommon.

## Conclusion

Weekly injections of Hylan G-F 20 starting at 1 week after surgery delayed cartilage degeneration after meniscectomy in rats. Synovitis induced by meniscectomy was not improved by Hylan G-F 20. Foreign bodies were observed in the synovium after the Hylan G-F 20 injection.

### Ethics approval

This study was approved by the local ethics committee (The Animal Experimental Committee, Tokyo Medical and Dental University, Japan; study number 0160119).

### Availability of data and materials

The de-identified data and material supporting the findings in this study could be provided through direct contact to the main author (KY).

### Consent for publication

Not applicable.

## Abbreviations

OA: Osteoarthritis
OARSI: Osteoarthritis Research Society International
PBS: Phosphate buffered saline
